# Phenotypic Disease Network-Based Multimorbidity Analysis in Idiopathic Cardiomyopathy Patients with Hospital Discharge Records

**DOI:** 10.3390/jcm11236965

**Published:** 2022-11-25

**Authors:** Lei Wang, Ye Jin, Jingya Zhou, Cheng Pang, Yi Wang, Shuyang Zhang

**Affiliations:** 1 State Key Laboratory of Complex Severe and Rare Diseases, Department of Cardiology, Peking Union Medical College Hospital, Chinese Academy of Medical Sciences & Peking Union Medical College, Beijing 100730, China; 2 Medical Research Center, State Key Laboratory of Complex Severe and Rare Diseases, Peking Union Medical College Hospital, Chinese Academy of Medical Sciences & Peking Union Medical College, Beijing 100730, China; 3 Department of Medical Records, Peking Union Medical College Hospital, Chinese Academy of Medical Sciences and Peking Union Medical College, Beijing 100730, China; 4 Collaborating Center for the WHO Family of International Classifications in China, Beijing 100730, China

**Keywords:** multimorbidity analysis, idiopathic cardiomyopathy, network, cosine index, associate rules analysis

## Abstract

Background: Idiopathic cardiomyopathy (ICM) is a rare disease affecting numerous physiological and biomolecular systems with multimorbidity. However, due to the small sample size of uncommon diseases, the whole spectrum of chronic disease co-occurrence, especially in developing nations, has not yet been investigated. To grasp the multimorbidity pattern, we aimed to present a multidimensional model for ICM and differences among age groups. Methods: Hospital discharge records were collected from a rare disease centre of ICM inpatients (*n* = 1036) over 10 years (2012 to 2021) for this retrospective analysis. One-to-one matched controls were also included. First, by looking at the first three digits of the ICD-10 code, we concentrated on chronic illnesses with a prevalence of more than 1%. The ICM and control inpatients had a total of 71 and 69 chronic illnesses, respectively. Second, to evaluate the multimorbidity pattern in both groups, we built age-specific cosine-index-based multimorbidity networks. Third, the associated rule mining (ARM) assessed the comorbidities with heart failure for ICM, specifically. Results: The comorbidity burden of ICM was 78% larger than that of the controls. All ages were affected by the burden, although those over 50 years old had more intense interactions. Moreover, in terms of disease connectivity, central, hub, and authority diseases were concentrated in the metabolic, musculoskeletal and connective tissue, genitourinary, eye and adnexa, respiratory, and digestive systems. According to the age-specific connection, the impaired coagulation function was required for raising attention (e.g., autoimmune-attacked digestive and musculoskeletal system disorders) in young adult groups (ICM patients aged 20–49 years). For the middle-aged (50–60 years) and older (≥70 years) groups, malignant neoplasm and circulatory issues were the main confrontable problems. Finally, according to the result of ARM, the comorbidities and comorbidity patterns of heart failure include diabetes mellitus and metabolic disorder, sleeping disorder, renal failure, liver, and circulatory diseases. Conclusions: The main cause of the comorbid load is aging. The ICM comorbidities were concentrated in the circulatory, metabolic, musculoskeletal and connective tissue, genitourinary, eye and adnexa, respiratory, and digestive systems. The network-based approach optimizes the integrated care of patients with ICM and advances our understanding of multimorbidity associated with the disease.

## 1. Introduction

Cardiomyopathies are structurally and/or functionally aberrant myocardial diseases that occur without the existence of coronary artery disease, hypertension, valve disease, or congenital heart disease [1,2]. After ruling out cases with known or suspected causes, primary cardiomyopathies are most likely caused by genetics. Other recently discovered causes involve sarcomeres, Z-discs, sarcoglycans, the cytoskeletal complex, transcription factors, calcium handling, the nuclear envelope, potassium and sodium channels, heat shock chaperones, and mitochondria [3]. However, up to 50% remain absent of a definite cause and are summarized as idiopathic cardiomyopathy (ICM) [4]. As a rare cardiovascular disease, the overall incidence of ICM has been estimated as 3.83 and 2.94 for every 10,000 person-years, in males and females, respectively, of Taiwan [5], while the overall prevalence is 31.5 per 100,000 in Japan [6]. In European countries, the prevalence of all dilated cardiomyopathies in the idiopathic population is unknown; however, it might be as high as 51%, equating to yearly incidence rates of 5–8 per 100,000 [7]. In the United States, the ICM was determined to be roughly 76 per 10,000 diabetic patients [8], according to their hospital discharge rate. In most cases, heart transplantation is necessary after medical therapy fails [9]. Nearly 40% of symptomatic patients [10] require heart transplantation or die within the first two years.

Genetic abnormality is the main intrinsic factor for ICM, and its pathogenesis remains heterogeneous. The analysis of co-occurrence diseases may help in the discovery of pathogeneses. For instance, patients with skeletal muscle myopathy showed an ICM-like phenotype that has been discovered to share pathogenesis with ICM [11]. Co-occurrence (comorbidities) has become an increasingly important topic among rare diseases [12], as a better understanding of comorbidities might provide help in case detection with limited samples, and even the underlying pathogenesis might lead to an overall improvement in health prognosis. The comorbidities of ICM have been previously discussed, apart from heart failure, and other chronic medical conditions including hypertension [13], obesity, stroke [14], diabetes mellitus [15,16], and autoimmune rheumatic diseases [17,18,19]. A recent investigation into rare disease was restricted by a rare sample and mostly focused on a few prevalent illnesses with prevalence statistics and a correlation analysis. Nonetheless, the comorbid patterns of ICM were addressed by clinical guidelines.

Complex networks have recently been developed for a proliferation of medical research in comorbidity pattern discovery [20,21,22]. Hidalgo et al. [23] utilized a network analysis to track the development of the illness. Zhou et al. [24] applied the phenotypic disease network for comorbidity patterns of ischemic heart disease. A network-based analysis was used in a case-control cohort study for the examination of COPD comorbidities [25]. Based on a comorbidity network, hepatocellular carcinoma comorbidity patterns were studied by Mu et al. [26]. ICD-10 diagnosis codes contained in hospital discharge records (HDR) are the foundation of these studies and offer great potential to assist in understanding the nature of comorbidities. However, none of these studies utilized HDR and network theories in the comorbidity pattern discovery of ICM. In this study, we set out to thoroughly investigate the comorbid status of hospitalized patients with ICM across the whole range of chronic diseases, in order to reveal the previously unrecognized relationships among diseases.

## 2. Material and Methods

### 2.1. Study Population 

Based on the Hospital Quality Monitoring System (HQMS) [27] database, the hospital discharge records (HDRs) from tertiary hospitals and rare disease centres in Beijing were gathered for retrospective research. Each HDR comprised 346 factors, including information about the department’s diagnosis, practices, and costs, among others. During the entire study period (1 January 2012–31 December 2021), 805 inpatients were diagnosed with ICM, with 1036 HDRs selected from a total of 850,381 hospitalizations. The inpatients with ICM were selected by both ICD code [I42.0, I42.4, I42.5, I42.8, I42.9, Q24.8] and a specialist in cardiology. The unique patient identification, sex, age, date, admission and discharge departments, primary discharge diagnosis, and up to 40 secondary diagnoses were all included in each. All diagnoses were specified by the International Classification of Diseases, 10th Revision (ICD-10). The frequency of the primary discharge diagnosis in ICM cases is listed in Supplementary Table S1, which is mainly composed of the diagnosis in I42 and Q24. We also selected the one-to-one control—from patients without a rare disease diagnosis—with propensity score methods by hospitalizations without a rare disease diagnosis, and year of birth (±2 years), gender, discharge time, and department.

### 2.2. Comorbidities and Comorbid Burden

To distinguish acute and chronic disorders, the ICD-10 codes’ three-digit format was used [28,29]. Some codes represented non-diseases or general symptoms but not disease. Therefore, we excluded codes in chapters XIX, XX, and XXI. Rare illnesses (prevalence under 1%) were also eliminated from further calculation in order to achieve more accurate estimates [30,31]. Following the aforementioned criteria, the ICM and control groups, respectively, identified 71 and 69 chronic disorders with a prevalence of over 1% as comorbidities, in which 57 were common (Supplementary Table S2). The number of comorbidities diagnosed in each patient throughout the research period is known as the comorbid burden.

### 2.3. Comorbidities Enrichment

The strength of chronic illness co-existence with ICM inpatients was assessed by the odds ratio (OR) of each comorbidity and the related 95% confidence intervals (95% CIs), when compared to controls. The Bonferroni adjustment was used to account for repeated testing. *p*-values under 0.05/71 indicate the statistical significance of OR. Disorders with higher odds (ORs over 1.5 with *p* values under 0.05/71) occurring in inpatients were referred to as enrichment comorbidities.

### 2.4. Network Analysis

#### 2.4.1. Multimorbidity Network Generation and Network Properties Calculation

Multimorbidity networks focused on chronic illnesses with over 1% prevalence for more accurate and trustworthy estimations. To discover the changes among ages, networks were generated in terms of these seven age strata: ≤19, 20–29, 30–39, 40–49, 50–59, 60–69, ≥70 years.

A network is made up of a collection of nodes connected by edges. Each node indicates a chronic condition (ICD-10 codes of three digits), whose colour and size correspond to the ICD-10 category. Edge denotes the co-existence of a comorbidity. For the disease pair coexisting by chance alone, its likelihood is often lower than severe comorbidity pairs. Typically, the higher the comorbid strength of a disease pair, the lower the probability of co-existence by chance alone [23,32]. Common comorbid strength evaluation approaches include the Pearson correlation coefficient and relative risk (RR; computed in Equation (1)). The Salton cosine index (SCI; computed in Equation (4)) might, in contrast, be immune to sample size and solely take co-occurrences and the incidence of multimorbidity into account [33]. Thus, the edges are strengthened by the SCI, and the cut-off of SCI [34] restricts the number of edges.

(1) $$RR_{ab}=\frac{n_{ab}*N_{total}}{n_{a}*n_{b}}$$

(2) $$\phi_{ab}=\frac{n_{ab}*N_{total}-n_{a}*n_{b}}{n_{a}*n_{b}*\left(N_{total}-n_{a}\right)*\left(N_{total}-n_{b}\right)}$$

(3) $$t=\frac{\phi_{ab}*\sqrt{n-2}}{\sqrt{1-\phi_{ab}^{2}}},\;n=max\left(n_{a},n_{b}\right)$$

(4) $$SCI_{ab}=\frac{n_{ab}}{\sqrt{n_{a}*n_{b}}}$$

The Kolmogorov–Smirnov (KS) test was used to determine whether or not the network’s degree distribution is power-law-like, as with other structural property indexes [35], in which the closeness centrality calculates the disease’s proximity to other chronic illnesses. So, the likelihood of a disease occurring in conjunction with other diseases in fewer stages increases with the closeness centrality of its network. The betweenness centrality refers to the number of shortest pathways across the diseases. In a network, a disease’s chance of building bridges to other diseases increases with its betweenness centrality.

#### 2.4.2. Disease Statuses—Central, Hubs, or Authorities

By taking into account the edge weights, the PageRank [36], hub, and authority values were derived to differentiate the node status in the network. With a higher PageRank value, the disease had a more “central” network status [37], as with authority. Hub [38] could be labelled as “important” for subsequent diagnoses. The nodes designated as central, hubs, and authorities, respectively, were those with the top 10 percentile above each value. By calculating all three values in the network for the 7 age groups of each ICM and control group, the appearance time of each chronic condition was displayed.

#### 2.4.3. Association Rules Mining Specifically for Heart Failure Occurrence in ICM

**Figure 1:**
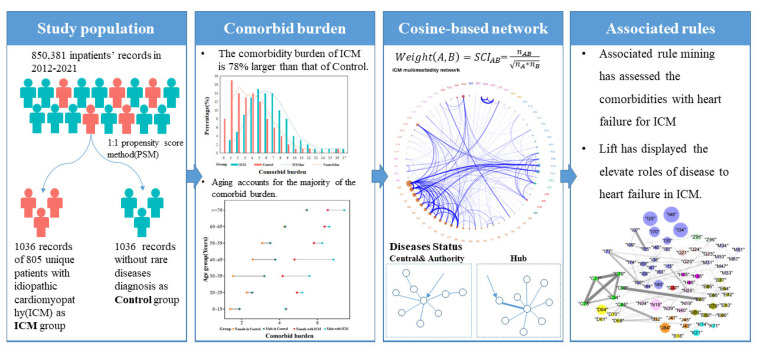
Research Flow chart. The whole process of comorbidities analysis was displayed, including population selection, comorbidity definition, network settlement, and association with heart failure.

**Figure 2:**
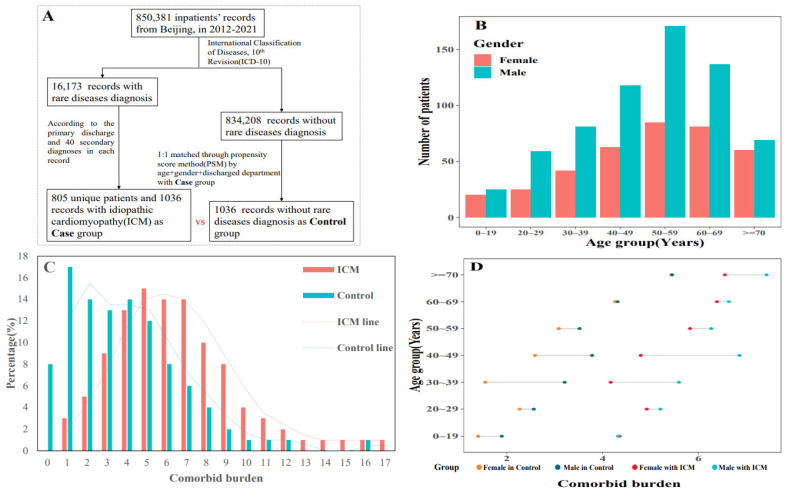
Participants’ selection and baseline information description. (**A**) performed the inclusion and exclusion process of participants;(**B**) the gender distribution in both selected groups; (**C**) the comorbid burden in each group and ICM obtained heavier burden; (**D**) the variation of comorbid burden among seven age groups and between gender.

Heart failure (HF) is the worst prognosis of ICM. We aimed to discover which of the chronic conditions are more likely to co-occur with HF in ICM patients and adopted association rule mining (ARM) [39] for the analysis. The Apriori algorithm is a popular itemset method for mining association rules. Support (the number of incidences of disease A and disease B among all patients) and confidence are used to assess the association rules (the number of occurrences of disease A co-occurring with disease B). According to the performance of validation illnesses, support >0.01 and confidence >0.5 were used in this study. For each association rule, the lift is also an important associated value. Lift calculates the ratio between the observed co-occurrence frequency P(A, B) and the predicted co-occurrence frequency P(A) P(B) when A and B are independent, in order to determine the importance of a rule’s support P(A, B). This criterion means less if the ratio is near to one. If the ratio is larger than one, A and B are positively associated; otherwise, they are negatively connected. R software (www.r-project.org/ accessed on 17 October 2022) was used for all statistical analysis, network constructs, and visualizations (version 3.5.1). The whole analysis process is listed in Figure 1 with the participants’ selection process in Figure 2A.

## 3. Results

### 3.1. Chronic Diseases and Comorbidities Burden

With 1036 HDRs, 805 ICM inpatients survived the trial term. At enrolment, the sample’s average age was 51 years, and 36.29% of the participants were female (Figure 2B). The mean number of comorbidities (5.86 vs. 2.45) and the proportion of patients with at least two comorbidities (96.72% vs. 58.49%) were greater in ICM when compared to controls (Figure 2C). In seven age groups, males possessed a greater comorbid burden than females in both ICM and control groups, while the burden increased among age groups. (Figure 2D). Fifty-seven chronic illnesses were statistically more likely to co-occur with ICM patients than controls among the 89 comorbidities with a prevalence of over 1% in cases. Table 1 shows the top 20 most common comorbidities, with over half of ICM cases comorbid with heart failure (I50, 77.51%), four in ten having complications, and ill-defined descriptions of heart disease (I51, 38.61%), and three in ten with atrial fibrillation and flutter (I48, 27.9%). In the meanwhile, comorbidities with a lower prevalence but a higher risk of co-occurring in ICM patients compared to controls remained. The prevalence of essential (primary) hypertension (I10), for example, was lower in ICM patients (prevalence = 28.67%, 95% CI: 25.93–31.53%), but it was more likely to co-occur in controls (OR = 0.58, 95% CI: 0.48–0.7). 

### 3.2. Properties of Age-Specific Multimorbidity Networks

**Figure 3:**
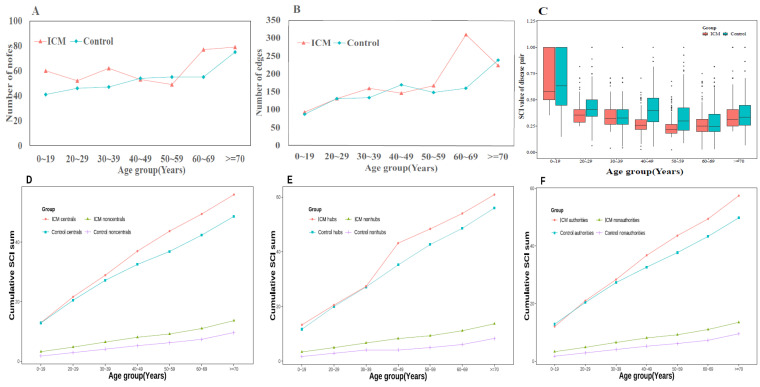
The feather of age-specific comorbidity network. (**A**) The variation of nodes in age-specific networks in both groups; (**B**) The variation of edges in age-specific networks in both groups; (**C**) the average of Salton cosine index in different age groups, ICM has lower SCI value than control groups in all age groups; (**D**) the cumulative SCI sum of central nodes in age groups; (**E**) the cumulative SCI sum of hub nodes in age groups; (**F**) the cumulative SCI sum of authority nodes in age groups, all “typical” nodes possessed stronger connectivity than the other “non-typical” nodes.

The varying trend of structural property indexes revealed the global structure of the network as well as chronic illnesses with a higher co-occurrence rate (Figure 3). The chronic diseases included in each age group are listed in Supplementary Table S3. By settling the multimorbidity networks across age strata, the number of nodes ranged from 49 to 79, and 41 to 75 in ICM and control groups, respectively. Edges were distributed from 93 to 311, and 87 to 240, respectively. The distribution of both edges and nodes increased according to the age groups, except for two subgroups, including 40–49 and 50–59 (Figure 3A,B). SCI score was calculated for each disease pair in seven age subgroups of both the ICM and control groups. The mean SCI value in the ICM group was lower than that in the control group (Figure 3C). The decreasing trend was present in the first five age groups, while the mean SCI values in the other two age groups increased. The higher value of SCI identified stronger disease connections. The 0–19 age subgroup was low in edges but each disease pair was strongly connected. Furthermore, illness progression by age was assessed by examining the connectivity trajectories of each disease in the other part of Figure 3. Compared with the control group, ICM had higher connectivity according to the three indexes, including central, hub, and authority. The cumulative SCI sum of the central nodes in both groups was elevated among age subgroups; however, the ICM had a gentle slope in 50–59 and 60–69 subgroups (Figure 3D), as with hub and authority (Figure 3E,F). The “typical” nodes possessed stronger connectivity than the other “non-typical” nodes; for example, the cumulative SCI sum of the central nodes was higher than that of the non-central nodes in both the ICM and control groups, which conforms to the network topology.

The topological features of each network are listed in Table 2. Generally, the multimorbidity networks in the ≥70 age subgroups were sparser, and the density increased with increasing age. The maximum diameter of both groups was the 60–69 age group, with 7 and 10 in ICM and control groups, respectively. In the centrality assessment of the multimorbidity network for the ICM group, the average closeness, betweenness, and degree centrality values were in descending order among age subgroups, besides the ≥70 subgroup. The degree and average neighbour degree in ICM were significantly higher than that of the middle-aged (50–69 age subgroups) control group, while significantly lower in the 0–19 age subgroup in both indexes. (Wilcoxon test, both *p* < 0.05).

### 3.3. Nodes Status in Age-Specific Network

**Figure 4:**
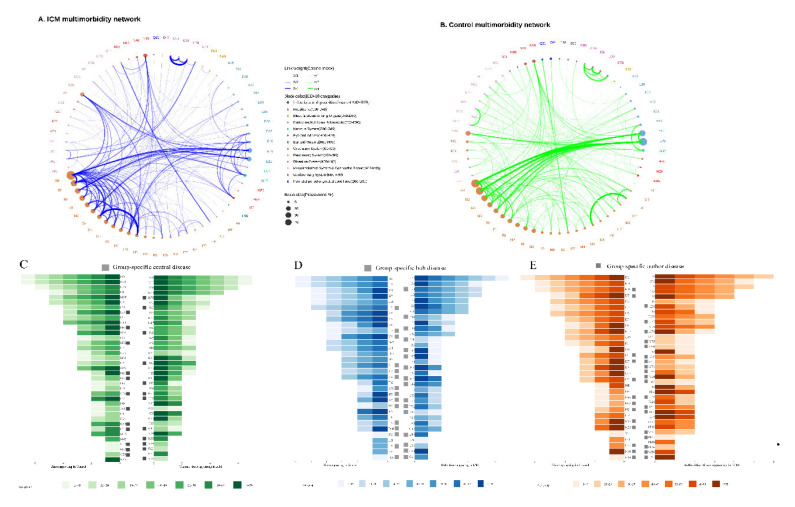
Comorbidity networks, central diseases, hubs, and authorities. (**A**) contained the network of ICM group(**B**) contained the network of control groups, the size of nodes represents the prevalence of diseases and width of edges stand for the correlative strength between diseases by the SCI value of each connection; (**C**) Central nodes in age-specific network. The central diseases were defined as the top 10 percentiles with PageRank in each network; (**D**) Hub nodes in age-specific network. The hub diseases were defined as the top 10 percentiles with hub value in each network;(**E**) Authority nodes in age-specific network. The authority diseases were defined as the top 10 percentiles with authority value in each network and have the similar effect with central nodes.

Figure 4A,B depicts the ICM and control multimorbidity networks, respectively. The connections in the ICM network were more complicated than those in the control network, based on the frequency and comorbidity strength of both networks. For ICM, the connections were concentrated in the circulatory system while connected with the metabolic (non-insulin-dependent diabetes mellitus [E11] and disorders of lipoprotein metabolism and other lipidaemias [E78]); neoplasm (malignant neoplasm of colon [C18], secondary malignant neoplasm of respiratory and digestive organs [C78], and secondary malignant neoplasm of other sites [C79]); eye and adnexa (H35); and genitourinary (disorders resulting from impaired renal tubular function [N25]) systems. Additionally, connections with liver disease (K76) were strengthened with metabolic diseases (E16, E66, and E78), sleep disorders (G47), retinal disorders (H35), and atherosclerosis (I70), while the range of comorbidities expanded to include goitre (E04) and circulatory diseases (I07, I10, I25, I34, I36, I50, I51). The most comorbid disease pair in ICM was cardiomyopathy (I42) to heart failure (I50), while, excluding the cardiomyopathy-related diagnoses, the disease pair with the highest SCI value was nonrheumatic mitral valve disorders (I34) to nonrheumatic tricuspid valve disorders (I36). Additionally, the connections in ICM occurred earlier than in the control; for instance, in ICM, heart failure (I50) and its complications (I51) occurred in the 0–19 subgroup and ≥40 years old in the control; atherosclerosis (I70) and aortic aneurysm and dissection (I71) were highly connected in almost all adults (≥20 years old) in ICM and appeared ≥50 years old in the control; atherosclerosis (I70) and liver disease (K76) co-existed in ICM patients ≥40 years old and ≥70 years old in the control; hyperfunction of the pituitary gland (E22) and sleep disorders (G47) exhibited strong comorbid strengths in adults from 20 to 60 years old and only in the control between 40 to 50 years old; and obesity (E66) and lipoprotein metabolism disorders (E78) connected in 0–19-year-old patients with ICM and only in adults in the control. (See details of connections in each network in Supplementary Table S4 for clinicians’ convenience in searching conditions of interest, as well as obvious and non-obvious comorbidity associations).

Three indices, including hubs, central, and authority, were used to identify disease status in the network. The top five hub diseases of ICM included metabolic (E11, E78) and circulatory diseases (I25, I34, and I50). The key diagnoses based on PageRank were essentially matched authoritative diagnoses in aggregate, with liver disease (K76) and heart failure and complications (I50, I51) in both top-five lists. The roles of each disease in the network are listed in Supplementary Tables S5–S7. The time appearance of the age subgroups is visualized in Figure 3C–E, in which 33 and 32 hubs were identified by all age subgroups with 17 diseases in common. Sixteen ICM-specific hubs include malignant neoplasm (C04, C80), thyroiditis (E06), metabolism disorder (E83, E89), hearing loss (H90), rheumatic tricuspid valve (I07), other heart disease (I44, I47, I48, I49), arthrosclerosis and arterial embolism (I70, I74), emphysema and bronchiectasis (J43, J47), and intestinal malabsorption (K90). For the 17 common hubs, iodine-deficiency hypothyroidism (E02), pituitary gland (E22), lipidaemia (E78), and occlusion and stenosis of the arteries (I65) appeared earlier in the 0–19 age subgroup with higher effects in 60–69-year-olds in ICM. Six chronic diseases were defined as both ICM-specific central and authorities, including sinusitis and bronchiectasis (J32, J47); fibrosis, cirrhosis, and inflammatory liver (K74, K75); scoliosis (M41); and contracted kidney (N26). For the common authority diseases, atrial fibrillation and flutter (I48) were identified as the authority with stronger (in 0–19 and 60–69 age subgroups) and larger (in 50–59 age subgroup) effects on ICM. Interstitial pulmonary (J84)-affected patients with ICM over 60 years old, while affecting those under 20 years old in the control. For the common central diseases, arthrosclerosis with complications (I51, I70), spondylosis (M47), and impaired renal tubular resulting (N25) disease covered more age subgroups of ICM.

Based on association rules analysis and patients with ICM, 26 rules were selected to indicate the comorbidities for heart failure. With increasing order of lift value and the heart failure in consequents status, diabetes mellitus and metabolic disorder (E11, E78), sleeping disorder (G47), liver (K76), circulatory (I10, I25, I27, I34, I36, I44, I47, I48, I49, I51, I70), renal failure (N18) could elevate the appearance of heart failure, which result have been listed in Table 3.

## 4. Discussion

### 4.1. Principal Findings

We constructed multimorbidity networks with EHR-based data from all inpatients with ICM. The networks for patients in control groups were generated following the same steps. Based on the comorbidity frequency, the comorbidity burden of ICM was 78% larger than that of the controls, in which the intricate interactions became increasingly intense over >50 years old. A data-derived complex network was applied for central diseases detection and the multimorbidity patterns analysis, which not only revealed connections in both the ICM and control groups, but also discovered their time appearance by age subgroups analysis. The comorbidities of ICM were concentrated in the circulatory, metabolic, musculoskeletal and connective tissue, genitourinary, eye and adnexa, respiratory, and digestive systems. Finally, with ARM, the comorbidities and their patterns of heart failure in patients with ICM were selected. Our findings show that using network-based algorithms to routinely gather healthcare data may provide a mechanism to better screen for and uncover complex relationships between chronic illnesses and rare diseases.

### 4.2. Comorbidity Diseases and Disease Pairs in Patients with ICM

The inclusion of a wide range of chronic disorders allowed for a thorough investigation of ICM’s chronic comorbidities. Comorbidities were fairly varied among ICM patients, with a few diseases having a high frequency and many others being less comorbid (e.g., 27.5% of comorbidities with a prevalence of >1%). ICM inpatients were twice as likely as controls to have a co-occurrence of 11 diseases (ORs > 2 with *p*-values according to the Bonferroni adjusted threshold). These are complex, system-level illnesses that impact a wide range of physiological and biomolecular processes, and hence, co-occur with a wide range of other disorders. Although the comorbidities of patients with ICM remained unknown according to the limited sample size of rare diseases, the network-based comorbidities analysis for cardiovascular diseases [24,30,31,40] has long existed. A cross-sectional study [21] on 34,099 discharged patients in Mexico discovered that cardiovascular disease (CVD) comorbidity networks were significantly localized in prominent conditions such as cardiac arrhythmias, heart failure, chronic renal disease, hypertension, and ischemic disorders. Another cross-sectional investigation examined the IHD comorbidity pattern, and disorders of the circulatory system, chronic renal failure, gastritis and duodenitis, and other metabolic diseases were detected. According to the results of the networks, we discovered that CVDs, including hypertension, were not limited to ICM inpatients (I10) [41], atrial fibrillation and flutter (I48) [42], ischemic diseases (I25) [21], heart failure and its complications (I50 and I51) [43], which is consistent with prior study findings. With a larger comorbidity load, a complete examination of the co-occurrence of physical and mental comorbidities is required to better understand ICM and ease therapeutic management.

Diseases of the circular system are included in the ICM-specified illness (e.g., heart failure and complications, atrial fibrillation and flutter, cardiac arrhythmias, atrioventricular and left bundle-branch block, nonrheumatic tricuspid and mitral valve disorders, and pulmonary heart diseases), chronic renal failure, and sleep disorders. The affection of the latter two diseases to cardiomyopathy may result in the following two ways. Renal failure [44] affects cardiomyopathy by altered cardiac histone H3 epigenetics and fosters cardiomyocyte hypertrophy in type 2 diabetes. Sleep-disordered [45] breathing is a commonly seen symptom in patients with idiopathic cardiomyopathy, especially with dilated cardiomyopathy, which may result in obesity. Moreover, the association of ICM with diabetes mellitus (E11) was higher than that of the control, however, without significance (OR = 1.08, 95% CI: 0.87–1.33), as with cerebral infraction (I63), as OR = 1.04 (95%CI 0.62–1.74). These might be primarily attributed to the unavoidable selection bias in previous retrospective studies. According to the data-driving methods, networks, and ARM, the other negative associations of ICM with hypertension (I10) were also found to strongly connect to both ICM and heart failure. As the network is an undirected graph, the aforementioned correlations are not causal relationships [34]. As a result, patients with ICM may struggle to discover a precise cause and outcome through this network, but it may provide information on co-occurring disorders in each age range for the purpose of therapeutic guidance.

Our dataset included hospitalizations of all ages, and hence, includes information regarding illnesses that were prevalent and particular to age groupings. For example, illness pairs that occurred throughout life were ICM-linked (e.g., nonrheumatic mitral or tricuspid valve disorders with complications of heart disease and lipo-protein metabolism disorders co-occurring with diabetes mellitus). Other ICM-specific disease pairs included liver diseases [46] and associations with lipidaemia and lipoprotein metabolism and the circulatory system. The study’s discovery of lifetime comorbid illness pairings may provide ideas for improving integrated management in multimorbidity patients.

### 4.3. Age-Specific Diseases Pairs and Hub, Central, Authority Disease Defined

Ageing was responsible for the majority of comorbidities in ICM patients, which is comparable to how other chronic illnesses develop (e.g., ischemic heart disease patients [24], and chronic obstructive pulmonary disease [47]). We discovered that age influenced the comorbidity pattern in ICM patients. ICM patients’ physical comorbidity load increased with age, and the comorbidity network became more intricate. Some couples had a higher comorbid strength but only occurred in a specific age range. Obesity and lipidaemia and lipoprotein metabolism were specified for ICM patients <49 years old. Adolescents with severe obesity [48] had obtained higher risk in their adulthood with cardiomyopathy, heart failure, cardiovascular mortality, and all-cause death; for instance, cardiac arrhythmias (I49) were connected with scoliosis (M41) in both the 0–19 age group and all ICM groups, in which patients with congenital and idiopathic scoliosis had an increasing incidence of congenital cardiac abnormalities [49] (e.g., cardiomyopathy). Other age-specified connections included coagulation defects (D68) and cerebral infraction (I63) in the 20–29 age group; purpura (D69) and liver diseases (K74) in 30–39 year olds; rheumatoid arthritis (M06) and osteoporosis (M81) in 40–49 year olds; malignant neoplasm of the mouth (C04) and secondary malignant neoplasm of the lymph nodes (C77) in 50–59 year olds; secondary malignant neoplasm (C79) and aortic aneurysm and dissection (I71) in 60–69 year olds; and heart failure (I50) and (N18) in the ≥70 age groups. As a result, it is recommended that the therapy of young adult groups (20–49 years) of ICM patients be a priority for illnesses arising from decreased clotting function, auto-immune-attacked digestive and musculoskeletal system problems. For the coagulation defects, its crosstalk with complement activation was a promoter of cardiac dysfunction, especially for cardiomyopathy [50]. For the middle-aged (50–60 years) and older (≥70 years) groups, malignant neoplasm and circulatory problems are expected to increase attention to which cancer treatment, especially anthracyclines and trastuzumab [51], may be cardiomyopathy related. Thus, the data-driven identification of co-occurring illnesses may be valuable in generating prospective theories for coexisting diseases [52,53] (for example, sharing the same gene, having shared risk factors, and exhibiting a consistent temporal development trend) and their age disparities [28].

By identifying the central, authority and hub diseases, the status was even more clear. The central and authority diseases share the same meaning in the network; they can all be interpreted as those that are in the centre of the networks and more likely to correlate with multimorbidity or lead to multimorbidity. Heart failure (HF) demonstrated its authority position in the network as an independent mortality predictor of patients with ICM [54]. An additional ICM-specified ARM for HF was analysed with diabetes mellitus and metabolic disorder [55], sleeping disorder, liver disease, circulatory disease, and renal failure [56], as HF risk elevators. These are cardiac and non-cardiac comorbidities for both ICM and HF, indicating the strong interaction effects among HF and its comorbidities with mortality than each independent effect [54]. The hub diagnoses (those with a high likelihood of eventual multi-morbidity) may be tightly linked to others. The hubs include metabolic (E), eye and adnexa, circulatory, and respiratory diseases [57], which are all comorbidities of HF with interactive effects on mortality [58]. As a result, clinical investigations of the indicated illness state may be beneficial in improving prevention measures and healthcare policies [59]. The data-driven finding of co-occurring illnesses [60], particularly when based on a large population with a high-quality healthcare database, may influence disease management.

### 4.4. Limitations

Our study contained certain limitations that should be mentioned. First, given the undirected weighted graph, it was impossible to determine the causation and interrelationships of the observed correlations; these associations will be further identified by fundamental experiment studies. Second, the sample size was limited when compared with research for comorbidities pattern analysis. For a rare disease, the sample size has long been a problem in the research setting [61,62]; however, the SCI score assessed the correlations among diseases with cosine as the weight to eliminate the bias effect of sample size. In future studies, we will further enlarge the sample size in each age group to strengthen the correlations. Finally, the multimorbidity network was only constructed with the HDR, as it is a fully constructed dataset with ICD-10 codes for a unified diagnosis. Without the help of the longitudinal follow-up, it was hard to reveal the causal relationship among the diseases. To further strengthen the network effects, in the future, the networks will include admission, discharge records, and follow-up records by avoiding the underestimation of the comorbidity burden.

### 4.5. Study Strength

In conclusion, our comprehensive investigation of comorbidities in hospitalized ICM patients in China offers an overview of the disease’s chronic physical and mental comorbidity in routine inpatient care. ICM inpatients had a 78% higher comorbidity load than matched controls, and the comorbidity network became more complicated with age. The comorbidities of ICM were concentrated in the circulatory, metabolic, musculoskeletal and connective tissue, genitourinary, eye and adnexa, respiratory, and digestive systems. Diabetes mellitus and metabolic disorders, sleeping disorders, liver, circulatory, and renal failure could elevate the appearance of heart failure. Finally, the data-driven network and cosine-based connection employed in our study may examine all multimorbidity relationships at the population level for ICM, which might be used within healthcare data sets in different situations.

## Figures and Tables

**Table 1:** The comorbidity prevalence of both ICM and control groups.

ICD Matched Name of Diseases	ICD-10-Code	Prevalence in ICM	Prevalence in Control	OR (95%CI)
Heart failure	I50	77.51 (74.84, 80.02)	20.56 (18.14, 23.15)	13.32 (10.8, 16.42) *
Nonrheumatic mitral valve disorders	I34	18.92 (16.58, 21.44)	2.32 (1.49, 3.43)	9.84 (6.38, 15.18) *
Nonrheumatic tricuspid valve disorders	I36	10.14 (8.36, 12.14)	1.45 (0.81, 2.38)	7.68 (4.44, 13.28) *
Other pulmonary heart diseases	I27	15.64 (13.48, 17.99)	2.8 (1.88, 4)	6.44 (4.29, 9.65) *
Hyperfunction of pituitary	E22	4.15 (3.02, 5.55)	0.68 (0.27, 1.39)	6.37 (2.85, 14.22) *
Atrioventricular and left bundle-branch block	I44	13.8 (11.76, 16.05)	2.61 (1.72, 3.77)	5.98 (3.93, 9.12) *
Complications and ill-defined descriptions of heart disease	I51	38.61 (35.63, 41.65)	10.33 (8.54, 12.34)	5.46 (4.31, 6.91) *
Atrial fibrillation and flutter	I48	27.9 (25.18, 30.74)	10.14 (8.36, 12.14)	3.43 (2.69, 4.37) *
Other cardiac arrhythmias	I49	21.62 (19.15, 24.26)	9.65 (7.92, 11.62)	2.58 (2, 3.33) *
Chronic renal failure	N18	13.61 (11.58, 15.85)	5.89 (4.53, 7.5)	2.52 (1.84, 3.45) *
Sleep disorders	G47	8.11 (6.52, 9.94)	3.57 (2.53, 4.89)	2.38 (1.6, 3.54) *
Paroxysmal tachycardia	I47	0.14 (0.12, 0.16)	0.1 (0.09, 0.12)	1.44 (1.1, 1.87) *
Other diseases of liver	K76	0.16 (0.14, 0.19)	0.13 (0.11, 0.16)	1.27 (0.99, 1.61)
Non-insulin-dependent diabetes mellitus	E11	0.22 (0.19, 0.24)	0.21 (0.19, 0.24)	1.04 (0.84, 1.28)
Atherosclerosis	I70	0.13 (0.11, 0.15)	0.13 (0.11, 0.15)	1.04 (0.8, 1.34)
Essential (primary) hypertension	I10	28.67 (25.93, 31.53)	40.93 (37.91, 43.99)	0.58 (0.48, 0.7) *
Disorder of lipoprotein metabolism and other lipidaemias	E78	21.33 (18.87, 23.95)	34.65 (31.75, 37.64)	0.51 (0.42, 0.62) *
Chronic ischaemic heart disease	I25	19.59 (17.22, 22.14)	32.53 (29.68, 35.48)	0.51 (0.41, 0.62) *
Acute myocardial infarction	I21	0.77 (0.33, 1.52)	11 (9.16, 13.07)	0.06 (0.03, 0.13) *
Angina pectoralis	I20	0.29 (0.06, 0.84)	13.9 (11.85, 16.16)	0.02 (0.01, 0.06) *

Notes, * represents α=0.05, with Bonferroni *p* value <$\frac{\alpha }{89}$.

**Table 2:** The topological properties of networks in total and age subgroups.

Group	Sample Size	Diameter	Density	Average Path Length	Average Closeness	Average Neighbour Degree	Average between	Average Degree	Minimum SCI Value
**ICM**									
All	1036	5	0.13	2.26	0.02	13.28	24.21	7.11	0.03
0~19	45	4	0.05	1.65	0.33	3.36 *	1.93	3.1 *	0.15
20~29	84	4	0.1	1.97	0.14	5.53 *	6.3	5.04	0.06
30~39	123	6	0.08	2.16	0.07	6.39	10.5	5.16	0.04
40~49	181	6	0.11	2.34	0.04	7.05	13.4	5.55	0.06
50~59	256	5	0.14	1.97	0.04	8.61 *	10.14	6.86 *	0.09
60~69	218	7	0.11	2.56	0.01	9.73 *	34.69	8.08 *	0.03
>=70	129	6	0.07	2.24	0.04	7.03	13.34	5.7	0.07
**Control**									
All	1036	7	0.14	2.25	0.02	11.97	25.69	8.53	0.02
0~19	39	5	0.11	1.73	0.13	5	3.3	4.24	0.35
20~29	87	6	0.13	1.92	0.13	7.2	6.76	5.7	0.25
30~39	122	6	0.12	1.94	0.09	6.94	6.91	5.7	0.04
40~49	181	5	0.12	2.12	0.08	7.39	11.63	6.3	0.03
50~59	259	6	0.1	2.37	0.04	6.62	14.93	5.42	0.02
60~69	219	10	0.11	2.75	0.02	6.85	23.05	5.85	0.03
>=70	129	6	0.09	2.16	0.06	7.37	12.86	6.4	0.2

Notes, * represents α=0.05, with *p* value < α.

**Table 3:** The associated rule mining for heart failure in patients with ICM.

Antecedents ICD Matched Disease Name	Antecedents ICD-Code	Consequents ICD-Code	Antecedent Support	Support	Confidence	Lift	Leverage	Conviction
{Complications and ill-defined descriptions of heart disease; Other pulmonary heart disease}	{‘I51′, ‘I27′}	{‘I50′}	0.09	0.085	0.946	1.221	0.015	4.183
{Other pulmonary heart disease}	{‘I27′}	{‘I50′}	0.156	0.146	0.932	1.203	0.025	3.312
{Nonrheumatic mitral valve disorders}	{‘I34′}	{‘I50′}	0.189	0.174	0.918	1.185	0.027	2.755
{Nonrheumatic mitral valve disorders; Complications and ill-defined descriptions of heart disease}	{‘I34′, ‘I51′}	{‘I50′}	0.151	0.138	0.917	1.183	0.021	2.699
{Complications and ill-defined descriptions of heart disease; Atrial fibrillation and flutter}	{‘I51′, ‘I48′}	{‘I50′}	0.116	0.106	0.917	1.183	0.016	2.699
{Complications and ill-defined descriptions of heart disease; Nonrheumatic tricuspid valve disorder}	{‘I51′, ‘I36′}	{‘I50′}	0.084	0.076	0.908	1.172	0.011	2.446
{Sleep disorders}	{‘G47′}	{‘I50′}	0.081	0.073	0.905	1.167	0.011	2.361
{Nonrheumatic tricuspid valve disorder}	{‘I36′}	{‘I50′}	0.101	0.091	0.895	1.155	0.012	2.147
{Nonrheumatic mitral valve disorders; Nonrheumatic tricuspid valve disorder}	{‘I34′, ‘I36′}	{‘I50′}	0.092	0.082	0.895	1.154	0.011	2.137
{Complications and ill-defined descriptions of heart disease; Chronic ischaemic heart disease}	{‘I51′, ‘I25′}	{‘I50′}	0.091	0.081	0.894	1.153	0.011	2.114
{Essential (primary) hypertension; Chronic ischaemic heart disease}	{‘I10′, ‘I25′}	{‘I50′}	0.094	0.083	0.887	1.144	0.01	1.983
{Atrioventricular and left bundle-branch block}	{‘I44′}	{‘I50′}	0.138	0.122	0.881	1.137	0.015	1.892
{Essential (primary) hypertension; Disorders of lipoprotein metabolism and other lipidaemias}	{‘I10′, ‘E78′}	{‘I50′}	0.1	0.088	0.875	1.129	0.01	1.799
{Atherosclerosis}	{‘I70′}	{‘I50′}	0.13	0.114	0.874	1.128	0.013	1.786
{Complications and ill-defined descriptions of heart disease; Essential (primary) hypertension}	{‘I51′, ‘I10′}	{‘I50′}	0.105	0.092	0.872	1.124	0.01	1.751
{Complications and ill-defined descriptions of heart disease}	{‘I51′}	{‘I50′}	0.386	0.334	0.865	1.116	0.035	1.666
{Chronic ischaemic heart disease}	{‘I25′}	{‘I50′}	0.196	0.169	0.862	1.112	0.017	1.631
{Atrial fibrillation and flutter}	{‘I48′}	{‘I50′}	0.279	0.24	0.862	1.112	0.024	1.625
{Disorders of lipoprotein metabolism and other lipidaemias}	{‘E78′}	{‘I50′}	0.213	0.183	0.86	1.109	0.018	1.603
{Complications and ill-defined descriptions of heart disease; Disorders of lipoprotein metabolism and other lipidaemias}	{‘I51′, ‘E78′}	{‘I50′}	0.093	0.079	0.854	1.102	0.007	1.542
{Chronic renal failure}	{‘N18′}	{‘I50′}	0.136	0.116	0.851	1.098	0.01	1.51
{Non-insulin-dependent diabetes mellitus}	{‘E11′}	{‘I50′}	0.218	0.185	0.85	1.096	0.016	1.495
{Paroxysmal tachycardia}	{‘I47′}	{‘I50′}	0.142	0.12	0.844	1.088	0.01	1.437
{Complications and ill-defined descriptions of heart disease; Other cardiac arrhythmias}	{‘I51′, ‘I49′}	{‘I50′}	0.111	0.094	0.843	1.088	0.008	1.437
{Non-insulin-dependent diabetes mellitus; Essential (primary) hypertension}	{‘I10′, ‘E11′}	{‘I50′}	0.092	0.077	0.842	1.086	0.006	1.424
{Other diseases of liver}	{‘K76′}	{‘I50′}	0.164	0.137	0.835	1.078	0.01	1.365

Note: The ICD code “I50” represents heart failure.

## Data Availability

The datasets used and/or analysed during the current study are available from the corresponding author on reasonable request.

## Supplementary Materials

The following supporting information can be downloaded at: https://www.mdpi.com/article/10.3390/jcm11236965/s1, Table S1: The frequency of the primary discharge diagnosis in ICM cases; Table S2: Chronic comorbidities of patients in both ICM and control groups; Table S3: Chronic comorbidities of patients in each of seven ages groups; Table S4: Connections in age-specific networks; Table S5: Authorities nodes of networks; Table S6: Central nodes of networks; Table S7: Hub nodes of networks.
